# Examining associations between upsizing, downsizing, workplace offensive behaviors and sickness absence due to common mental disorders – a longitudinal cohort study

**DOI:** 10.1186/s12889-025-25203-9

**Published:** 2025-11-17

**Authors:** Maria Wijkander, Rebecka Holmgren, Hugo Westerlund, Linda L. Magnusson Hanson

**Affiliations:** 1 Division of Psychobiology and Epidemiology, Department of Psychology, Stockholm University, Stockholm, 10691 Sweden; 2 Division of Insurance Medicine, Department of Clinical Neuroscience, Karolinska Institutet, Stockholm, Sweden; 3 Department of Public Health Sciences, Stockholm University, Stockholm, Sweden

**Keywords:** Bullying, Expansion, Mental disorders, Personnel downsizing, Sick leave, Violence

## Abstract

**Background:**

Prior research on health and work-related consequences of workforce restructuring have shown mixed findings. This study seeks to examine if organizational upsizing and downsizing is prospectively associated with sickness absence due to common mental disorders (SA-CMD) and/or with exposure to workplace offensive behaviors (i.e. workplace bullying and workplace violence/threats of violence), and assess if workplace offensive behaviors mediate any association between organizational upsizing/downsizing and SA-CMD.

**Methods:**

This study used a prospective design, combining self-reported survey data from the Swedish Longitudinal Occupational Survey of Health and national register data covering the period 2010–2018 on 10 358 employed individuals. Logistic regression with generalized estimating equations was used to examine the associations between organizational upsizing/downsizing, offensive behaviors, and sickness absence due to common mental disorders. To examine the role of offensive behaviors in the association between exposure to upsizing/downsizing and sickness absence due to common mental disorders, mediation analysis was performed.

**Results:**

We found that upsizing, but not downsizing, was significantly associated with a higher risk of workplace violence/threats of violence (ORadj: 1.2, 95% CI 1.0-1.3). However, neither upsizing nor downsizing was significantly associated with workplace bullying or with subsequent SA-CMD and the mediation analysis indicated no mediation.

**Conclusions:**

Although this study did not find a link between organizational upsizing/downsizing and later sickness absence due to SA-CMD, it suggests that upsizing may be associated with certain types of workplace offensive behaviors. Further research is needed to explore the mechanisms associated with these dynamics.

**Supplementary Information:**

The online version contains supplementary material available at 10.1186/s12889-025-25203-9.

## Background

Organizational restructuring is common in modern work life, and the rate of change has been reported to increase in both private and public sectors [1]. While organizational changes usually aim to increase productivity and/or reduce costs, it has been argued that this may be at the expense of employees’ mental health and well-being, and their ability to work [2, 3]. However, empirical evidence on mental health-related consequences of organizational changes is relatively scarce, and characterized by substantial methodological heterogeneity, making it hard to draw any firm conclusions [4–6]. One particular form of organizational change is workforce restructuring, which can include both downsizing (reductions in staff) and upsizing (expansions in staff). Compared to other forms of organizational change, staff reduction has been suggested to have more severe negative consequences [7]. Several studies have found prospective associations between downsizing and poor mental health, purchase of psychotropic medication and increased risk of sickness absence [8–11]. Meanwhile, other studies have either not found any statistically significant associations between downsizing and sickness absence or reported that downsizing reduces the risk of sickness absence [12, 13]. Several studies further report that the associations vary depending on the size of the downsizing and prior health status of the employees, and whether short- or long-term effects are examined [9, 14, 15]. Health outcomes after exposure to upsizing are less examined, and existing studies report mixed findings. For example, Westerlund et al. [15] found that accumulated exposure to moderate upsizing was associated with a decreased risk of hospital admissions for somatic and psychiatric illnesses, while repeated exposure to major expansions (defined as an increase of staff of more than 18%) was linked to an increase in hospital admissions, and an increased risk of subsequent long-term sickness absence. In another study, increases in staffing, corresponding to 20% or more, reduced the risk of later sickness absence [14]. Clearly, regarding upsizing, more research is needed to clarify any presence and direction of association with health [4].

One way in which organizational restructuring is hypothesized to affect mental health and work ability is by altering the psychosocial working environment [16], including the workplace social climate and the social relations at work [1, 11]. Focusing on the latter, studies have linked exposure to organizational restructuring, including organizational downsizing and redundancies, to decreased social support [1] and to several psychosocial work factors including low social support, workplace bullying and verbal aggression [17]. On the other hand, Kivimäki et al. found no significant association between downsizing and social support from supervisors or colleagues [11]. In several studies, organizational restructurings have also been linked to increased occurrence of exposure to offensive behaviors such as workplace bullying [18, 19]. Findings regarding the relationship between organizational restructuring and work-related violence or aggression are mixed. While some studies have identified links to violence and workplace aggression, others have found no such association [17, 20, 21].

Exposure to workplace offensive behaviors (e.g. workplace bullying and workplace violence), can negatively impact the mental health and work ability of the targeted individual. Prior studies, including our own work, have found that exposure to workplace bullying (multiple exposure to negative social acts over time [22]) and violence or threats of violence (any unacceptable behaviors or threats thereof aiming at resulting in, or are likely to result in physical, psychological, sexual or economic harm [23]) is linked to subsequent indicators of poor mental health, such as depression [24–26], purchase of psychotropic medication [25, 27, 28] and sickness absence due to common mental disorders [29–31]. With the exception of one cross-sectional study [17], exposure to offensive behaviors has not yet been studied as a potential intermediate factor on the pathway from organizational changes to poor mental health, and none has to our knowledge focused on sickness absence as the end point.

Therefore, the aim of the present study is two-fold. First, we aim to examine if organizational restructuring (i.e. upsizing and downsizing) is prospectively associated with mental health related-sickness absence and with exposure to workplace offensive behaviors (i.e. workplace bullying and workplace violence/threats of violence). Second, we aim to examine if, and to what extent, workplace offensive behaviors mediate the association between organizational restructuring and mental health-related sickness absence. Increased knowledge about direct and indirect associations between organizational restructuring and mental health-related sickness absence can inform organizations on how to retain employees healthy and in work after organizational changes.

## Methods

The present study was performed with a prospective design, using a combination of self-reported survey data and data from national registers.

### Study sample and data

The Swedish Longitudinal Occupational Survey of Health (SLOSH), is a follow-up of Swedish Work Environment Surveys (SWES), and thereby based on an approximately nationally representative sample of the working population in Sweden, aged 16–64 years. SLOSH was initiated in 2006. In 2016, SLOSH consisted of 40 877 individuals from SWES 2003–2011 who had received questionnaires biennially (added to SLOSH successively between 2006 and 2014) [32]. There are two different versions of the SLOSH questionnaire, one for people in paid work (≥ 30% of full-time) and one for individuals working less than 30% or not working at all. Depending on the respondents’ situation at the time of data collection, they answer the survey suited to their form of activity. The in-work survey covers a range of organizational and psychosocial work characteristics as well as social factors and indicators of health and wellbeing. These are measured through a combination of validated scales and items used in SWES questionnaire or developed for SLOSH (see www.slosh.se for more information). Data from SLOSH-surveys can be linked to several national registers through personal identification numbers. Prior findings show that respondents to SLOSH are more likely to be women, married, older and have a university degree [32].

**Fig. 1:**
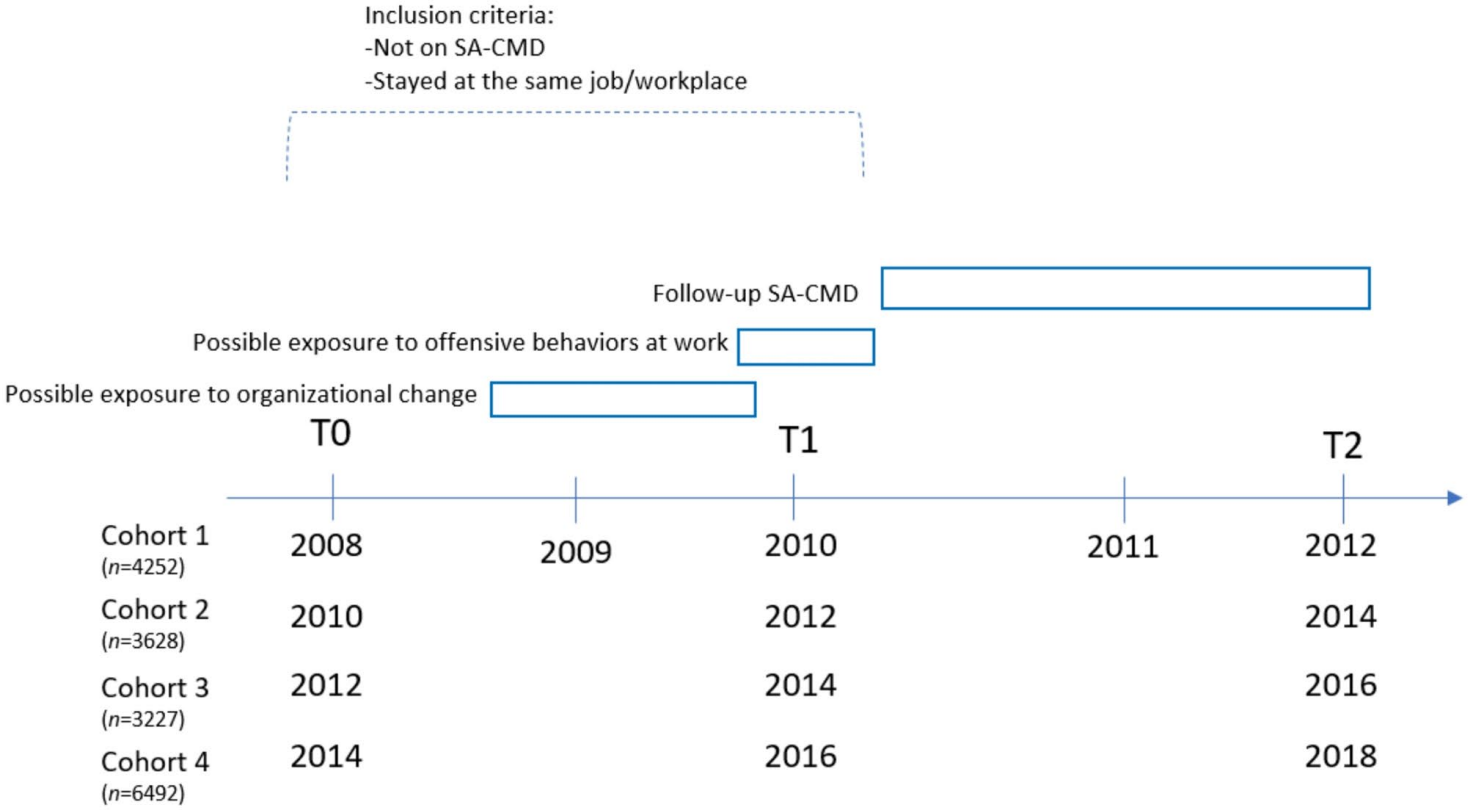
Study design and time line of data collection for the four nested cohorts, including number of observations in each cohort

Our source population consisted of individuals who had participated in at least two consecutive survey waves of the in-work version of SLOSH starting with 2008 to 2014. We arranged the data as four sub-cohorts with their baseline year (T0) being either 2008, 2010, 2012 or 2014 and the follow-up year (T1) being either 2010, 2012, 2014 or 2016. See Fig. 1 for study design and timeline of data collection. The SLOSH survey is distributed in spring each wave. To maximize the number of study observations individuals could contribute with several observations. This means that if an individual had participated in two consecutive in-work surveys several times (e.g. 2008–2010 and 2014–2016), they were included twice (counted as two observations). The data from these four sub-cohorts was pooled.

**Fig. 2:**
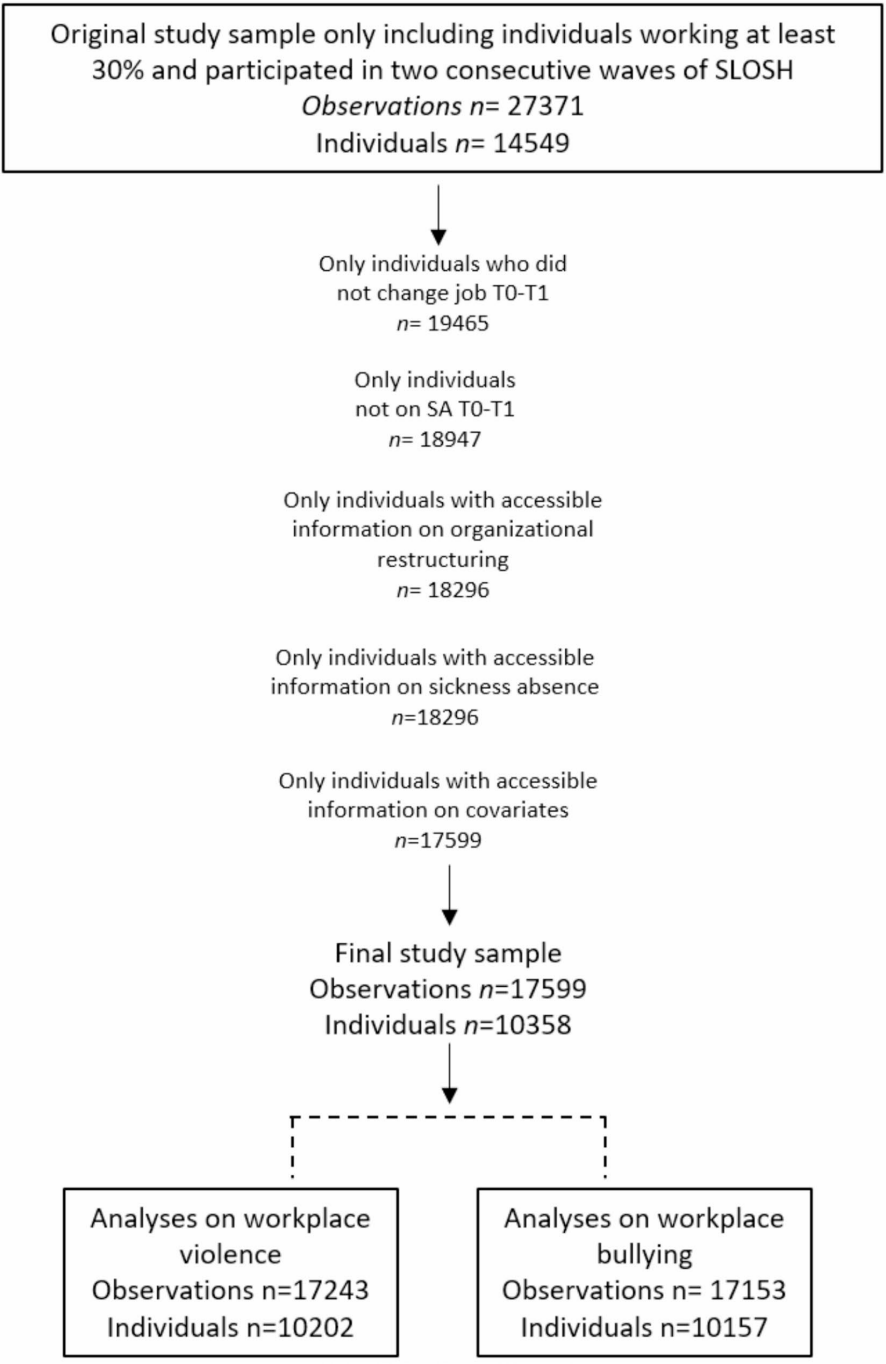
Inclusion criteria of the final full study sample and the number of observations/individuals included in the analyses of work-place violence and workplace bullying

The sample was restricted to participants who worked at the same establishment between T0 and T1 (based on data retrieved from national registers) and who did not have any spell of SA-CMD during this period. We excluded participants with missing data on organizational restructuring or missing data on adjustment covariates. This resulted in a study sample of 17 599 observations, corresponding to 10 358 unique individuals. Further, when analyzing exposure to workplace violence/threats of violence or exposure to workplace bullying respectively, only complete cases (no missing data on these covariates at either T0 or T1) were included. Consequently, the size of the analytical samples varied between 17 153 and 17 243 observations. See Fig. 2 for flowchart of sampling process.

### Organizational restructuring

Register-based data, derived from the Statistics on Dynamics of Enterprises and Establishments (DEE), was used as exposure measurement. The DEE contains organizational level information regarding structural changes of enterprises and establishments in Sweden, collected since 1986. Enterprises refers to the main organization, whereas establishments are registered locations for organizations (each enterprise can have one or more establishments). Using the LISA-register held by Statistics Sweden (SCB) [33], containing individual-level information regarding establishment, we were able to identify if an individual was working at an establishment that was undergoing structural changes across a 12-month period as registered in the DEE (November one year to November the next). In line with prior research, *downsizing* was defined as a reduction of staff of at least 8% and *upsizing* defined as an increase of staff of 8% or more [15]. Only individuals working at establishments that still existed in the subsequent year were included, thus, closures were not considered. We created a variable with three mutually exclusive categories: unexposed (working at an establishment with no or little staff change), exposed to upsizing, exposed to downsizing. This data was paired with SLOSH survey data using personal identification numbers.

### Sickness absence due to common mental disorders (SA-CMD)

Data on individual level sickness absence was derived from The Micro Data for Analyses of Social Insurance (MiDAS), again using individual personal identification numbers. MiDAS is a register held by the Social Insurance Agency of Sweden, containing information on all sickness absence and disability pension benefits in Sweden since the year 1994. Information regarding diagnoses is available from the year 2005. In Sweden, longer sickness absence spells must be accompanied by a medical certificate (issued at day 7 at the latest), including primary diagnosis. Benefits are generally given from day 15 of the sickness absence spell. Since the focus of the present study was on mental health-related sick leave, only sickness absence spells lasting > 14 consecutive days with the diagnostic codes F30-F39 (mood/affective disorders) or F40-F48 (somatoform disorders) (according to the tenth version of the International Statistical Classification of Disease and related Health problems (ICD-10)), were included. The variable was treated as a dichotomous variable, indicating presence/absence of any sick leave spell. We measured the outcome during a 2-year period, starting from the time point of survey return in T1.

### Exposure to offensive behaviors

Exposure to violence and/or threats of violence was assessed with one survey question in the SLOSH questionnaire, asking whether the respondent had been exposed to violence and/or threats of violence in their job role. Exposure to bullying was assessed in a similar way, with one question in the SLOSH questionnaire, asking whether the participant had *“been subjected to personal persecution in the form of unkind words or behaviors from superiors or fellow workers”*. The reference period of exposure varied slightly (currently exposed/exposed in the past 12/6 months) between survey years (see supplementary table S1). For both items, the response alternatives were: *“Yes*,* one or several times/week”*,* “Yes*,* one or several times/month”*,* Yes*,* sometimes during the last 6 months”*,* “No”.* Each measure was dichotomized (yes/no), with the first three response options all coded as *“Yes”.*

### Covariates

Covariates included questionnaire data on civil status (married or cohabiting/single) and having children living at home (yes/no) (see supplementary table S2) and registry-data (from Statistics Sweden and the LISA-register) on sex (male/female), age (≤ 35 years/36–55 years/≥56 years), educational level and line of business/industry. Participants were divided into four different categories depending on their level of education: maximum of nine years of compulsory education, upper secondary education, < 3 years of post-secondary education and ≥ 3 years of post-secondary education. Regarding line of business/industry, the participants were categorized into five groups, according to the Swedish Standard Industrial Classification (SNI); construction and manufacturing; retail, hotel and transportation, restaurants and real-estate; law, science, economics and technology; education, health care and social services; and culture, entertainment and leisure. The covariates were derived at T0. In addition, we derived information on any SA-CMD (yes/no) in the 5 years before T0. We further created a variable indicating baseline year.

### Statistical analysis

Analyses were performed using STATA version 18.

**Fig. 3:**
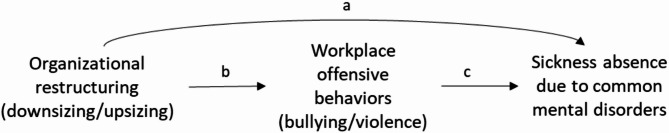
Graphical illustration of associations that will be examined

Figure 3 presents all examined associations. To address our first aim, we performed a series of logistic regression analyses using generalized estimating equations (GEE) with an exchangeable working correlation structure. GEE was chosen since it takes the intra-individual correlations between observations into account, thus accounting for the fact that individuals could contribute with multiple observations. Firstly, the association between exposure to organizational restructuring and SA-CMD was analyzed (a in Fig. 3) and secondly the associations between organizational restructuring and workplace bullying and organizational restructuring and workplace violence was examined respectively (b). The results from these analyses were also used for the second aim of the study – to assess the indirect association (mediation) between organizational restructuring and SA-CMD via bullying/violence. A statistically significant association between organizational restructuring and SA-CMD would indicate that the exposure of interest is a predictor of the outcome SA-CMD, which is one of the standard criteria for mediation according to the Baron and Kenny approach [34]. Similarly, a statistically significant association between organizational restructuring and bullying or violence would indicate that the exposure is a predictor of the potential mediator, which is another criterion for mediation according to Baron and Kenny. However, to assess if the additional criteria for mediation according to Baron and Kenny was fulfilled, namely that the mediator predict the outcome while adjusting for the exposure, we also performed an analysis investigating the association between workplace bullying and SA-CMD and workplace violence and SA-CMD (c). For all examined associations, a set of models, with increasing level of adjustment, were fitted. Covariates were identified through directed acyclic graphs (DAGs), drawn based on prior research. Different adjustments were thus applied for each examined association (these are presented in Table 1). For associations a (organizational restructuring and SA-CMD) the fully adjusted models included baseline year, education, line of business/industry and prior SA-CMD (before T0). Associations b (organizational restructuring and bullying/violence) were adjusted for baseline year, education, line of business/industry and prior offensive behaviors (at T0). The fully adjusted models for the associations c (bullying/violence and SA-CMD) were adjusted for baseline year, education, line of business/industry and prior SA-CMD (before T0) and additionally adjusted for sex, age, civil status and having children living at home, as well as for organizational change (upsizing/downsizing). Results from crude and adjusted models are shown (presented as odds ratios (OR) with 95% confidence intervals (CI)). When these analyses suggested that mediation could be present (i.e. in the case of finding statistically significant associations in all three analyses [34] or in the analyses of exposure-mediator and mediator-outcome associations [35]), we proceeded to examine mediation using causal mediation analysis (using the mediation command in STATA). In such mediation analysis, the total effect of organizational restructuring on SA-CMD is decomposed into the natural indirect effect (the effect of organizational restructuring on SA-CMD that operates via exposure to workplace bullying/violence) and the natural direct effect (the effect of organizational restructuring on SA-CMD that is not via bullying/violence) [35, 36]. All models were evaluated using 95% significance level (*p* < 0.05).

**Table 1:** Descriptive statistics, full study sample. Covariates measured at T0

Total observations		17,599
Exposure to organizational restructuring at T1		*n* (%)
	Exposure to upsizing	3408 (19)
	Exposure to downsizing	3363 (19)
	Stable (no restructuring)	10,828 (62)
Exposed to work-related violence at T1		
	Yes	1394 (8)
	No	16,027 (91)
	*Missing*	178 (1)
Exposed to work-related bullying at T1		
	Yes	1235 (7)
	No	16,128 (92)
	*Missing*	236 (1)
SA-CMD T1-T2		
	Yes	481 (3)
	No	17,118 (97)
Sex		
	Women	9790 (56)
	Men	7809 (44)
Age		
	< 35 years	982 (6)
	36–55 years	8127 (46)
	> 55 years	8490 (48)
Married or cohabiting		
	Yes	14,066 (80)
	No	3533 (20)
Children living at home		
	Yes	8582 (49)
	No	9017 (51)
Education		
	Compulsory 1	1318 (8)
	Upper secondary	7227 (44)
	< 3 years university	1271 (7)
	≥ 3 years university	7283 (41)
SNI (line of business)		
	1: construction and manufacturing	4270 (24)
	2: retail, hotel, transportation, restaurants, real-estate	3534 (20)
	3: law, science, economics, technology	1407 (8)
	4: education, health care, social services	7613 (43)
	5: culture, entertainment, leisure	775 (5)

## Results

### Descriptive statistics

Descriptive statistics of the sample is presented in Table 2. The sample consisted of more women than men, and the majority was either married or cohabiting. Most of the participants were above 36 years of age and the majority had an education level of either upper secondary school or had been studying on university level for three years or more. The largest proportion of the participants was employed within the healthcare or education sector.

**Table 2:** Results from GEE logistic regression models examining the associations between a) organizational restructuring and SA-CMD, b) organizational restructuring and workplace offensive behaviors and c) workplace offensive behaviors and SA-CMD

**Exposure**	**Outcome**	**Observations**	**Observations exposure**	**Observations outcome**	**% observations outcome**	**Model 1** **OR (95% CI) Crude**	**Model 2 ** **OR(95% CI ) Adjusted for baseline year, education and line of business**	**Model 3** **OR (95% CI) Additionally adjusted for prior SA-CMD**	
Unexposed	SA-CMD	17599	10828	299	3	Ref	Ref	Ref	
Upsizing			3408	95	3	1.0 (0.8-1.3)	1.0 (0.8-1.3)	1.0 (0.8-1.3)	
Downsizing			3363	87	3	0.9 (0.7-1.2)	1.0 (0.8-1.3)	1.0 (0.8-1.3)	
						**Model 1 **	**Model 2 **	**Model 3 **	
						**OR (95% CI) Crude**	**OR (95% CI) Adjusted for baseline year, education and line of business**	**Additionally adjusted for prior offensive behaviors**	
Unexposed	Violence	17243	10594	826	8	Ref	Ref	Ref	
Upsizing			3344	295	9	1.1 (1.0-1.3)	1.2 (1.0-1.3)	1.2 (1.0-1.4)	
Downsizing			3305	257	8	1.0 (0.9-1.1)	1.1 (0.9-1.2)	1.1 (0.9-1.3)	
Unexposed	Bullying	17153	10528	768	7	Ref	Ref	Ref	
Upsizing			3335	218	7	0.9 (0.8-1.1)	0.9 (0.8-1.1)	0.9 (0.8-1.1)	
Downsizing			3290	235	7	1.0 (0.8-1.1)	1.0 (0.9-1.2)	1.0 (0.9-1.2)	
						**Model 1**	**Model 2 **	**Model 3 **	**Model 4 **
						**OR (95%CI) Crude**	**OR (95%CI) Adjusted for baseline year, sex, age, marital status, children, education, line of business**	**OR (95%CI) Additionally adjusted for prior SA-CMD**	**OR ( 95%CI) Additionally adjusted for upsizing/downsizing**
Unexposed	SA-CMD	17243	15865	404	3	Ref	Ref	Ref	Ref
Violence			1378	69	5	2.0 (1.5-2.6)*	1.5 (1.1-1.9)*	1.4 (1.0-1.8)	1.4 (1.0-1.8)
Unexposed	SA-CMD	17153	15932	412	3	Ref	Ref	Ref	Ref
Bullying			1221	58	5	1.9 (1.4-2.5)*	1.8 (1.3-2.3)*	1.7 (1.3-2.3)*	1.7 (1.3-2.3)*

### Logistic regression analyses using generalized estimating equations

#### Exposure to organizational restructuring and subsequent SA-CMD

The upper part of Table 1 shows the results from logistic regression analysis using GEE for the association between organizational restructuring and SA-CMD. No statistically significant association between the exposure (either downsizing or upsizing) and the outcome (SA-CMD) was found.

#### Exposure to organizational restructuring and subsequent onset of exposure to workplace offensive behaviors

In the analyses of the associations between upsizing/downsizing and the potential mediating variable, a statistically significant association was found between exposure to upsizing and exposure to violence and/or threats of violence. In the most adjusted model, individuals working at workplaces undergoing upsizing had 1.2 times higher odds of experiencing subsequent exposure to workplace violence (OR_adj_: 1.2, 95% CI 1.0–1.4.). No other statistically significant associations were found (see middle part of Table 1).

#### Exposure to offensive behaviors and subsequent SA-CMD

Finally, in the analyses of the associations between the potential mediating variable (exposure to violence and/or threats of violence or exposure to bullying) and SA-CMD, we identified statistically significant associations in all models (see lower part of Table 1). The associations were attenuated in the more adjusted models.

#### Mediation analysis

Since we found a statistically significant association between exposure to upsizing and subsequent exposure to violence and/or threats of violence as well as a statistically significant association between exposure to violence and/or threats of violence and subsequent SA-CMD, two necessary criteria for mediation seemed to be fulfilled. Hence, we proceeded with mediation analysis decomposing the total effect of upsizing on the odds of SA-CMD into the direct and indirect effect, using exposure to violence and/or threats of violence as a mediator. In this analysis, exposure to violence and/or threats of violence was not found to mediate the association between exposure to upsizing and SA-CMD. See supplementary table S3.

## Discussion

We found that working in an organization undergoing upsizing, but not downsizing, was related to an increased risk of later exposure to workplace violence/threats of violence among men and women representative of the Swedish workforce. Neither upsizing nor downsizing was related to later exposure to workplace bullying. Our results further corroborated prior findings (based on the same source population) showing that exposure to workplace violence/threats of violence and workplace bullying was associated with subsequent sickness absence due to common mental disorders. However, the results did not suggest associations between organizational restructuring and later SA-CMD.

Consequently, the results of the present study could not confirm results of previous studies suggesting that there is an association between exposure to organizational restructuring and sickness absence. Previous studies have found that there is an association between exposure to downsizing and different kinds of negative outcomes such as mental health problems and sickness absence [4, 10]. The potential consequences of working at a workplace going through expansion/upsizing are less investigated. However, one previous study found that exposure to large expansions, with >18% of the employees affected, was associated to long term sickness absence [15]. In the present study organizational restructuring may have occurred quite some time before the sickness absence. A risk is that individuals with a certain vulnerability, who develop the outcome early on, have been removed and do not contribute to the follow-up [37]. This time lag could thus introduce selection, possibly affecting the association and may serve as a potential explanation to why the results of the previous study could not be corroborated in the present study. However, yet another study by Grønstad et al. (2019), investigating the association between exposure to unit-level upsizing and sickness absence found results indicating a reduced risk of sickness absence following unit-level upsizing [14], a result in line with the findings of the present study.

The results of our study did suggest an increased risk for exposure to offensive behaviors at work following organizational restructuring, at least in the form of workplace upsizing and exposure to violence and/or threats of violence. This increase in odds was seen even after adjusting for level of education and line of business which was identified as potential confounding variables. Since we did not differentiate between moderate upsizing of 8–18%, which in the earlier study by Westerlund et al. [15] was found to have beneficial effects, and upsizing >18%, which in the earlier study was associated with detrimental ones, it is possible that we have underestimated the negative effects of major upsizing events.

We did not observe an association between exposure to restructuring in the form of downsizing and an increased risk for exposure to offensive behaviors, and our results could thus not support the findings of a previous study by Baron and Neuman, who found a correlation between exposure to organizational changes (e.g. downsizing) and exposure to workplace aggression [20]. However, our study differs from theirs, both in terms of design (prospective vs. cross sectional) as well as in the measures used, which may explain the discrepancy in findings.

The inconsistency in findings between exposure to violence/threats and bullying could indicate that there may be different mechanisms at play. Prior studies, focusing on the perpetrator of offensive behaviors, have suggested that workplace violence/threats of violence are most likely to be carried out by external sources (such as clients or patients) [38], whereas workplace bullying more commonly is committed internally (either from colleagues or supervisors) [39]. Although not examined in our study, the fact that only workplace violence (not workplace bullying) increased at workplaces undergoing upsizing might suggest a “spill-over” effect of workplace upsizing on external sources. Another explanation could be that increased workplace violence could be the effect of lack of sufficient number of experienced staff during the upsizing process. We encourage future studies to further shed light on the mechanisms between workplace restructuring and offensive behaviors.

The results of the present study further indicated an association of exposure to both workplace violence and/or threats of violence or workplace bullying on SA-CMD. These results confirm the findings of several previous studies that have found links between exposure to an adverse psychosocial work environment and sickness absence [40], and specifically exposure to offensive behaviors at work and sickness absence due to CMD [29–31].

The results of the mediation analysis indicated that there was no direct effect of upsizing on SA-CMD, and no indirect effect (mediation) through violence and/or threats of violence. No indications of mediation by bullying were found neither for the associations between upsizing and SA-CMD nor downsizing and SA-CMD. In a recent study, Niedhammer et al. [17] found that both workplace bullying and workplace aggression mediated the association between organizational changes and self-reported depression. However, they applied a cross-sectional design, examined depression and not sickness absence and did not restrict the organizational change to instances of upsizing and downsizing, which may explain why our results differed.

Given the results found by Niedhammer et al. [17] and our other results suggesting associations between some forms of organizational restructuring and offensive behaviors, as well as between exposure to offensive behaviors and SA-CMD, we recommend further studies on this topic, possibly applying a different time lag between measurements.

### Strengths and limitations

The study is based on a fairly large representative sample of the Swedish working population, which can be considered a major strength. Its prospective design to a large extent ensures temporal order of events, and reduces the risks of reverse causation. However, due to DEE containing annual data only, a one month overlap between the period of organizational change and offensive behaviors could not be avoided.

Another strength of the present study is the use of both self-reported data as well as register-based data for measurement of exposure and intermediate variables. This strengthens the study’s validity since the use of merely self-reported data would include a risk of common method bias. Register-based data on mental health related outcomes further decreases possible information bias caused by social desirability or mood state [41].

However, the use of register-based data as exposure measurement implies a risk of misclassification. Data on organizational restructuring was collected at the establishment level (place where an organization operates), meaning that certain employees can be classified as exposed to an organizational change even though the particular part of the establishment where they are employed were not affected by the change. Similarly, employees in larger establishments could be classified as unexposed to restructuring even if their unit have undergone upsizing or downsizing (but this affected less than 8% of the total staff in the establishment). A further limitation when using register-based data on organizational restructuring is that the DEE-statistics only includes organizations of a certain size (very small organizations are not included), limiting the generalizability of our findings somewhat. We used self-reported information on offensive behaviors and chose to dichotomize the variables. This implies that we cannot rule out a risk of misclassification.

Other changes in working conditions and society that occurred during the same period of time, that potentially could have an impact on the outcome, such as shifts in the global economy and changes in laws and legislation regulating companies and working conditions, were not considered in the present study. This can be considered a limitation, lowering the external validity.

Several covariates and potential confounding variables were included in the present study, which can be considered a strength. Nevertheless, it cannot be ruled out that the observed associations in fact are explained by unmeasured confounding factors that was not possible to include, and this may be considered a limitation. We did not exclude individuals with baseline offensive behaviors, however adjusting for baseline offensive behaviors did not markedly alter the estimates.

Another potential limitation with the current study is that the cohort used might not be completely representative of the population. For example, it has been shown that the SLOSH cohort consists of a larger proportion of women as well as older individuals and individuals with higher educational attainment compared to the population in general [32], which might restrict generalizability of the findings. Another limitation is that the sample size prohibited us from performing sector-specific analysis. We encourage future studies to address this topic.

### Concluding remarks

Organizational changes and restructuring may be difficult to avoid in a rapidly changing world. While the results of this study did not show that downsizing or upsizing increased sickness absence due to CMD, they indicate that organizational restructuring might be a potential precursor to some types of offensive behaviors at work. This suggests that employers should invest in factors that can improve the social climate at the workplace, such as good social support from management including good communication, fairness and role clarity, to mitigate the potential negative impact on employees that a change process may have. Our study further supports that there is an association between exposure to workplace offensive behaviors and sickness absence due to common mental disorders, underlining the need for working with preventive measures. These results can be of use when planning major organizational restructuring.

## Supplementary Information

Supplementary Material 1.

Supplementary Material 2.

Supplementary Material 3.

## Data Availability

A strategy for data access has been developed to protect personal privacy of the participants. Requests for data for specific research projects or collaborations can be addressed to [data@slosh.se].

## Abbreviations

CMD: Common mental disorders
DEE: Dynamics of enterprises and establishments
GEE: Generalized estimating equations
ICD: The international statistical classification of disease and related health problems
LISA: The longitudinal integrated database for health insurance and labor market studies
MiDAS: Micro data for analyses of social insurance
OR: Odds ratio
SA: Sickness absence
SA-CMD: Sickness absence due to common mental disorders
SLOSH: The Swedish longitudinal occupational survey of health
SNI: The Swedish standard industrial classification
SWES: Swedish work environment surveys
