# Correction: Examining associations between upsizing, downsizing, workplace offensive behaviors and sickness absence due to common mental disorders – a longitudinal cohort study

**DOI:** 10.1186/s12889-026-26550-x

**Published:** 2026-02-24

**Authors:** Maria Wijkander, Rebecka Holmgren, Hugo Westerlund, Linda L. Magnusson Hanson

**Affiliations:** 1 Division of Psychobiology and Epidemiology, Department of Psychology, Stockholm University, Stockholm, 10691 Sweden; 2 Division of Insurance Medicine, Department of Clinical Neuroscience, Karolinska Institutet, Stockholm, Sweden; 3 Department of Public Health Sciences, Stockholm University, Stockholm, Sweden

**Correction: BMC Public Health 25**,** 3965 (2025)**

**https://doi.org/10.1186/s12889-025-25203-9**

Following publication of the original article [1], the authors reported an error in Table 2, the last column “Model 4” was inadvertently removed. The correct table is presented below.

**Table 1:** Results from GEE logistic regression models examining the associations between a) organizational restructuring and SA-CMD, b) organizational restructuring and workplace offensive behaviors and c) workplace offensive behaviors and SA-CMD

**Exposure**	**Outcome**	**Observations**	**Observations exposure**	**Observations outcome**	**% observations outcome**	**Model 1** **OR (95% CI)** **Crude**	**Model 2** **OR (95% CI)** **Adjusted for baseline year, education and line of business**	**Model 3** **OR (95% CI)** **Additionally adjusted for prior SA-CMD**	
Unexposed	SA-CMD	17599	10828	299	3	Ref	Ref	Ref	
Upsizing			3408	95	3	1.0 (0.8-1.3)	1.0 (0.8-1.3)	1.0 (0.8-1.3)	
Downsizing			3363	87	3	0.9 (0.7-1.2)	1.0 (0.8-1.3)	1.0 (0.8-1.3)	
						**Model 1** **OR (95% CI)** **Crude**	**Model 2** **OR (95% CI)** **Adjusted for baseline year, education and line of business**	**Model 3** **Additionally adjusted for prior offensive behaviors**	
Unexposed	Violence	17243	10594	826	8	Ref	Ref	Ref	
Upsizing			3344	295	9	1.1 (1.0-1.3)	1.2 (1.0-1.3)	1.2 (1.0-1.4)	
Downsizing			3305	257	8	1.0 (0.9-1.1)	1.1 (0.9-1.2)	1.1 (0.9-1.3)	
Unexposed	Bullying	17153	10528	768	7	Ref	Ref	Ref	
Upsizing			3335	218	7	0.9 (0.8-1.1)	0.9 (0.8-1.1)	0.9 (0.8-1.1)	
Downsizing			3290	235	7	1.0 (0.8-1.1)	1.0 (0.9-1.2)	1.0 (0.9-1.2)	
						**Model 1** **OR (95% CI)** **Crude**	**Model 2** **OR (95% CI)** **Adjusted for baseline year, sex, age, marital status, children, education, line of business**	**Model 3** **OR (95% CI)** **Additionally adjusted for prior SA-CMD**	**Model 4** **OR (95% CI)** **Additionally adjusted for upsizing/downsizing**
Unexposed	SA-CMD	17243	15865	404	3	Ref	Ref	Ref	Ref
Violence			1378	69	5	2.0 (1.5-2.6)*	1.5 (1.1-1.9)*	1.4 (1.0-1.8)	1.4 (1.0-1.8)
Unexposed	SA-CMD	17153	15932	412	3	Ref	Ref	Ref	Ref
Bullying			1221	58	5	1.9 (1.4-2.5)*	1.8 (1.3-2.3)*	1.7 (1.3-2.3)*	1.7 (1.3-2.3)*

The original article [1] has been updated. 
