# Supplementary material for: Examining associations between upsizing, downsizing, workplace offensive behaviors and sickness absence due to common mental disorders – a longitudinal cohort study
Source: BMC Public Health. 2025 Nov 17;25:3965. doi: 10.1186/s12889-025-25203-9 (PMC12621406; doi:10.1186/s12889-025-25203-9)
Supplement: Supplementary file 1 — Supplementary Material 1. [file 12889_2025_25203_MOESM1_ESM.docx]

*Supplementary table S1. Wording and response alternatives of item(s) measuring exposure to workplace violence/threats of violence and workplace bullying in SLOSH, by study year.*

| **SLOSH year(s)** | **Workplace violence/threats of violence** | **Workplace bullying** | **Response alternatives** |
| --- | --- | --- | --- |
| 2008* | Are you subjected to violence or threats of violence in your work? | Are you subjected to personal persecution in the form of unkind words or behaviors from your superiors or fellow workers? | Every day/Couple of days a week/One day a week/Couple of days a month/Sometimes during last 3 months/Once or twice during last 12 months/Not at all during the last 12 months |
| 2010^#^ | During the last 12 months, have you been subjected to violence or threats of violence in your work? | During the last 12 months, have you been subjected to personal persecution in the form of unkind words or behaviors from your superiors or fellow workers? | Yes/No |
| 2012,  2014,  2016^¤^ | During the last 6 months, have you been subjected to violence or threats of violence in your work? | During the last 6 months, have you been subjected to personal persecution in the form of unkind words or behaviors from your superiors or fellow workers? | Weekly/Monthly/Sometimes/No |

* These items are identical to the items in SWES 2003 and 2005.

^#^ These items were modified with regards to wording and response alternatives.

^¤^ The time frame for the exposure was changed and the response alternatives were changed to measure frequency of exposure.
