# Supplementary material for: Examining associations between upsizing, downsizing, workplace offensive behaviors and sickness absence due to common mental disorders – a longitudinal cohort study
Source: BMC Public Health. 2025 Nov 17;25:3965. doi: 10.1186/s12889-025-25203-9 (PMC12621406; doi:10.1186/s12889-025-25203-9)
Supplement: Supplementary file 2 — Supplementary Material 2. [file 12889_2025_25203_MOESM2_ESM.docx]

*Supplementary table S2. Wording and response alternatives of item(s) measuring sociodemographic characteristics in SLOSH.*

| **Sociodemographic characteristics** | Item | Response alternatives | SLOSH years |
| --- | --- | --- | --- |
| Civil status | Are you single or married/cohabiting? | Single/  Married or cohabiting | 2008-2016 |
| Children living at home | Do you have any children living at home?  Include children living with you at least half of the time. | Yes/No | 2008-2016 |
