# Supplementary material for: Examining associations between upsizing, downsizing, workplace offensive behaviors and sickness absence due to common mental disorders – a longitudinal cohort study
Source: BMC Public Health. 2025 Nov 17;25:3965. doi: 10.1186/s12889-025-25203-9 (PMC12621406; doi:10.1186/s12889-025-25203-9)
Supplement: Supplementary file 3 — Supplementary Material 3. [file 12889_2025_25203_MOESM3_ESM.docx]

*Supplementary table S3. The total effect of exposure to upsizing on the odds of SA-CMD, decomposed into direct effect and indirect effect using exposure to violence and/or threats of violence as a mediator.*

|  | OR | 95% CI |
| --- | --- | --- |
| TE | 0.999 | 0.791-1.261 |
| NDE | 0.997 | 0.790-1.260 |
| NIE | 1.001 | 0.993-1.010 |

TE; Total effect, NDE; Natural direct effect, NIE; Natural indirect effect, OR; Odds ratio, CI; Confidence interval, SA-CMD; sickness absence due to common mental disorders. The reference group consists of individuals who were not exposed to any workforce restructuring. The outcome model was adjusted for sex, age, marital status, children living at home, education, line of business, baseline year, previous exposure to violence/threats of violence and previous SA-CMD. The mediator model was adjusted for education, line of business and baseline year.
